# Hybridoma cell-culture and glycan profile dataset at various bioreactor conditions

**DOI:** 10.1016/j.dib.2016.10.003

**Published:** 2016-10-13

**Authors:** Hemlata Bhatia, Erik Read, Cyrus Agarabi, Kurt Brorson, Scott Lute, Seongkyu Yoon

**Affiliations:** a Department of Chemical Engineering, University of Massachusetts Lowell, Lowell, MA, USA; b Division II, Office of Biotechnology Products, Office of Pharmaceutical Quality, CDER, FDA, Silver Springs, MD, USA

## Abstract

This is an “11 factor-2 level-12 run” Plackett-Burman experimental design dataset. The dataset includes 11 engineering bioreactor parameters as input variables. These 11 factors were varied at 2 levels and 23 response variables that are glycan profile attributes, were measured “A Design Space Exploration for Control of Critical Quality Attributes of mAb” (H. Bhatia, E.K. Read, C.D. Agarabi, K.A. Brorson, S.C. Lute, S. Yoon S, 2016) [2].

**Specifications Table**

**Table:** 

Subject area	Biopharmaceuticals
More specific subject area	Bioprocess Development
Type of data	Excel file
How data was acquired	LC-MS/MS based assay
Data format	Raw
Experimental factors	11 bioreactor engineering parameters were varied at 2 levels to study their effect on product quality
Experimental features	The relationship between above-mentioned parameters and product quality attributes was studied.
Data source location	Center of Drug and Research, FDA, Silver Springs, MD
Data accessibility	Data is within this article

**Value of the data**

• This dataset presents the effect of cell-culture parameters on the product quality and can be used to study the relationship between bioreactor engineering parameters and protein quality.
• This might be useful for any research work including the glycan profiles of proteins with respect to any engineering parameters and can also be used to compare the data with similar studies.
• This data might be potentially used for any validation studies for the models, which involve product quality attributes.

## 1 Data

This is a Plackett–Burman experimental design data for glycan profile of protein produced by hybridoma cell line. Total number of 16 batches of data includes 12 test batches (numbered 1–12) and 4 control batches (numbered 13–16). This data includes 11-bioreactor operation parameters varied at 2 levels as input and 23 product quality attributes as output as explained in Table 1. [2].

The design of experiment used for this study is as explained in Transparency document, Appendix A and corresponding values for 23 glycan profiles for the protein for all batches are given in the data Transparency document, Appendix A.

## 2 Experimental design, materials and methods

The experimentation mentioned above was carried out in 2 phases. Each phase consists of 6 test batches and 2 control batches. All of the experiments were run at FDA (White Pak facility, Silver Springs, MD) using a murine IgG: K cell line. [1], [3].

The experimental methods used for glycan analysis were described in the reference [1]. The glycosylated antibody was purified from the cell culture fluid and glycans were released from the antibody with the help of PGNase buffer (New England Biolabs, Ipswich, MA), dialysis and Slide-A-Lyzer cartridge (Thermo Scientific, Pittsburgh, PA). The glycans were purified and dried using GlycoPrep H cartridge (Prozyme, Hayward, CA) and speedvac respectively. For the analysis, the instrument settings were based on a previous reference [4]. The glycans were labeled with 2-AB and purified before analyzing with LC-MS/MS based assay.

## Footnotes

Appendix A Supplementary data associated with this article can be found in the online version at doi:10.1016/j.dib.2016.10.003.
